# “We Have No ‘Visibly’ Trans Students in Our School”: Educators’ Perspectives on Transgender-Affirmative Policies in Schools

**DOI:** 10.1177/01614681221121522

**Published:** 2022-09-05

**Authors:** Wayne Martino, Kenan Omercajic, Jenny Kassen

**Affiliations:** 1 The University of Western Ontario, London, ON, Canada

**Keywords:** cisgenderism, cissexism, cisnormativity, critical trans politics, educators, gender-expansive education, transgender, trans-affirmative policies, trans students

## Abstract

**Background/Context:**

In Ontario, and Canada more broadly, anti-discrimination on the basis of gender identity and gender expression is enshrined in the Ontario Human Rights Code and the Canadian Charter of Rights and Freedoms, which have required schools to address trans inclusion. However, the ways in which educators understand or enact these policies, and whether they are even aware of them, remain largely underexplored.

**Purpose/Research Question/Focus of Study:**

Our purpose was to learn more about educators’ awareness and understanding of trans-inclusive policies in schools and the extent to which such policies were informing practice.

**Participants:**

While this research is based on survey data comprising 1,194 respondents, this article examines comments provided about trans-affirmative policy from 463 educators.

**Research Design:**

This study involves large-scale survey research conducted on 1,194 educators in Ontario K–12 schools; the survey was disseminated via social media and educational affiliates. We draw primarily on the qualitative data component of the survey, where educators provided detailed comments about and insights into trans-inclusive policies. We employed a reflexive approach to coding and thematic analysis to identify key themes.

**Findings/Results:**

Although our quantitative data depicted a favorable assessment of support for trans-affirmative policies—94% of respondents found their school’s policy to be very or somewhat relevant—our findings highlight a discrepancy between policy and practice, and a lack of commitment to addressing cisgenderist, cisnormative, and cissexist systems. The themes that emerged from our coding and analysis of the qualitative data were: (1) educators’ understanding of policy as accommodation; (2) individualized approaches to trans inclusion; (3) lack of administrative support and intervention; (4) the gap between policy and practice; (5) transphobic and cissexist resistance to supporting gender diversity; (6) the need for trans-affirming and gender-expansive curriculum, and (7) the problem of generalized approaches to equity and acceptance of diversity. In addition, we discuss several educator comments that raise important questions about race and the need for intersectional approaches to addressing equity and trans inclusion in schools.

**Conclusions/Recommendations:**

We advocate for a paradigm shift with respect to the necessity of employing a trans epistemological framework that addresses the need for gender-expansive education which focuses on the harmful effects of cisgenderism, cisnormativity, and cissexism in the education system. Central to addressing gender justice and trans marginalization in schools for all students, we conclude, is the need for policy makers to ensure accountability and budgetary allocation for the provision of resources and professional development for educators in schools.

## Introduction

In this article, we report on survey research involving 1,194 educators in Ontario K–12 schools that sought to generate knowledge and understanding of transgender inclusion and gender diversity in schools. We draw on the qualitative data component of the survey, through which educators had the opportunity to provide more detailed comments about trans-inclusive policies specifically, an important focus given the significant role that policy plays in supporting the well-being of trans^1^ students in schools. Peter et al. (2021), for example, found in their pan-Canadian climate study on homophobia, biphobia, and transphobia that “2SLGBTQ-focused [Two-Spirit, lesbian, gay, bisexual, transgender, queer/questioning] policy in schools acts as a protective factor for many 2SLGBTQ students” (p. 16) and has contributed to enhancing their mental health and well-being, as well as to reducing homophobic and transphobic harassment. Kosciw and colleagues’ (2020) school climate survey study in the United States revealed that students in schools with trans-inclusive polices were also less likely to experience discrimination and harassment and to feel a greater sense of belonging than their counterparts in schools without such policies or guidelines. In Ullman’s (2015) research in Australia, queer and trans students reported that teachers were more likely to address homophobic and transphobic harassment and to be “openly positive about same-sex attraction and gender diversity” (p. 35) in schools where inclusive policies that explicitly named gender and sexual diversity were in place. Ullman concluded that

these findings highlight an apparent relationship between institutional endorsement of inclusivity of sexuality and gender diversity, evidenced here via inclusive school policy directives which have been actively communicated to students, and a teaching staff that is more vocal on sexuality and gender diversity and related topics. (pp. 35–36)

Given this research, we wanted to learn more about educators’ awareness and understanding of trans-inclusive policies in schools and the extent to which such policies were informing practice. Our purpose was to generate knowledge and understanding of trans-inclusive policies in a context in which anti-discrimination, on the basis of gender identity and gender expression, is enshrined in the Ontario Human Rights Code (OHRC) and Canadian Charter of Rights and Freedoms (Bill C-16, 2016; Ontario Human Rights Commission, 2014; Martino et al., 2019). We bring a critical trans political lens to our analysis and framing of the study, which, as Spade (2015) noted, is less concerned with the law and policy, and more with the “administrative functions” and enactment of such policies. In this respect, we were concerned with learning more about educators’ awareness of such policies and how they conceptualized support for trans students in schools. We draw on trans studies scholars who provide insight into the institutional and systemic barriers, illuminating the impact of cisgenderism, cisnormativity, and cissexism^2^ in accounting for the failure of school staff to adequately support trans students and to educate about gender expansiveness in schools (Ansara, 2010; N. Kennedy, 2018; Serano, 2014). In addition, Ahmed’s (2012) theoretical and phenomenological work on how the language of diversity in educational organizations is deployed in performative ways that efface “the operation of systemic inequalities” (p. 53) also informed our analysis.

In what follows, we provide a review of the literature that focuses specifically on educators in schools and their responses to trans students. We illuminate how this body of work has both informed and contextualized our own contribution to the field. A brief overview of the policy context in Ontario is provided, followed by our theoretical framework and an overview of the study from which this data set is drawn. Finally, we provide an analysis and discussion of the data and address the implications of our study.

## Literature Review

There is a significant and growing body of research on educators’ response to the increasing visibility and presence of trans students in schools. For example, Payne and Smith (2014), in their research with district administrators, elementary school principals, classroom teachers, and student support staff in the United States, found that many of their participants felt afraid and unprepared to support trans students. The feelings of anxiety and lack of confidence incited by the presence of a trans child in their school were attributed to a lack of preparation and awareness in their training, highlighting the failure of university programs to educate about gender diversity and its implications for practice. Furthermore, the failure to provide any professional development (PD) at the district level, as well as clear policy guidelines, protocols, and directives for supporting trans students in schools, further exacerbated feelings of inadequacy and lack of preparedness. However, Smith and Payne (2016) note that attending such “Trans 101” training—which involved an “introduction to trans-specific vocabulary” (p. 39) and understanding the differences between sex, gender, and sexual orientation, as well as sharing research on gender and schooling, with a focus on their implications for policy and practice—did not necessarily lead to teachers’ willingness to embrace gender-expansive education.

Frohard-Dourlent’s (2016a) research in Canada provides additional insights into how educators make sense of their experiences in working with trans and gender-diverse students in schools. Their study extends Smith and Payne’s findings by providing additional knowledge about the ways that educators distanced themselves from the cisnormative systems of power in which they were implicated. Frohard-Dourlent revealed how educators downplayed both their nonintervention into harassment directed at trans students, reducing such behavior to merely struggling to fit in with one’s peers, and the harm enacted by their acts of misgendering students. These actions, Frohard-Dourlent (2016a) argues, were rationalized in ways that enabled educators to absolve themselves from “actively creat[ing] an inclusive environment” (p. 67) and addressing trans marginalization in schools. Educators also positioned themselves as “supportive rather than prejudiced” (p. 68), which resulted in a failure to recognize their “unintentional complicity in institutional practices that contribute to the maintenance of heteronormativity and gender conformity in schools” (p. 69).

Taylor et al. (2016) have also identified several significant gaps between educators’ beliefs and their practices, concluding that “participants at all levels [are] far more likely to approve of LGBTQ-inclusive education than to practice it” (p. 138)—a discrepancy that is, Bartholomaeus and Riggs (2018) suggest, related to “a skills gap.” These latter scholars claim that although teachers may feel more accepting of trans students, they may “still lack clarity about how to teach or support students” (Taylor et al., 2016, p. 138), and supportive teachers with knowledge may be hesitant to enact trans-affirmative education because of a lack of institutional support and fear of reprisal and community backlash (Ferfolja & Ullman, 2020; Morgan & Taylor, 2019). In their study with teachers in Canada, Meyer et al. (2016) identified the pervasiveness of transphobia in schools that has resulted in a high frequency of school transfers for trans students. Meyer et al. commented on the “balancing act that educators perform while trying to effectively navigate these complex issues with little formal training and institutional support” (p. 9; also see Morgan & Taylor, 2019).

This nexus of educators’ understanding of trans inclusion as it relates to practice underscores our contribution to the field in generating knowledge about the gap between trans-inclusive policies and practice in supporting trans students and gender-expansive education in schools. Leonardi and Staley (2018), for example, documented “the complex relationship between policy and practice” for school administrators in implementing their district’s “Guidelines for Supporting Transgender Students.” They investigated a PD program devoted to helping administrators “move beyond anti-bullying discourses to recognize and disrupt heteronormativity and binary thinking” (p. 758) in putting the guidelines into practice but acknowledged the limitations of such a queerly informed approach (Martino & Cumming-Potvin, 2018). Their findings reveal a more positive framing of administrators’ commitment to supporting gender diversity and trans inclusion as they “muddled through implementation” (Leonardi & Staley, 2018, p. 769) differently, albeit in ways that the authors constructed as a scaffolded and ongoing process of learning (see also Mangin, 2020). Luecke (2018), however, insists on “incorporat[ing] in-depth understandings of the gender nondiscrimination policy” (p. 279) in PD and in schools altogether, while Phipps and Blackall (2021) draw attention to the need for policy enactment that interrogates the embeddedness of cisgenderism and cisnormativity in school culture, which, their research revealed, is at the heart of trans marginalization in the education system (see also Riggs & Bartholomaeus, 2018). Our study adds to this body of work in the field by providing a comprehensive documentation of educators’ knowledge of trans-inclusive policies in Ontario, where school boards are required to abide by human rights legislation that recognizes gender identity and gender expression as protected grounds for anti-discrimination in schools.

## Ontario Policy Context

Ontario was one of the first jurisdictions in Canada to provide legislative support for trans and gender-diverse people, with many other provinces quickly following suit. Ontario introduced Toby’s Act in 2012 (Bill 33: Toby’s Act, 2012), which amended the OHRC to include both “gender identity” and “gender expression” as protected grounds against discrimination in areas such as education, housing, health care, and the workplace. Moreover, Ontario introduced this legislation five years before the Federal Government of Canada with the passing of Bill C-16 on June 19, 2017; this bill added the terms “gender identity or expression” to the Canadian Human Rights Act,^3^ along with the hate crimes provisions of the Criminal Code. In addition, the Accepting Schools Act (Bill 13: Accepting Schools Act, 2012) amended the Education Act to stipulate explicit protection for students from “bullying” based on their gender identity and gender expression. Moreover, the amendment underscored the importance of “a whole school approach” to being effective for queer and trans students “in creating a positive school climate and preventing inappropriate behaviour” (see also Luecke, 2018; Omercajic, 2022; Ullman, 2015). In fact, the Accepting Schools Act mandated that all publicly funded schools allow students to form GSAs (Gender and Sexuality Alliances) at their schools, a stipulation that has not been without controversy because of Catholic school board resistance^4^ (Martino et al., 2019).

It is noteworthy that one of the largest school boards in Ontario developed and released the first trans-affirmative policy of its kind in Canada in 2012. The policy reflected a legal requirement as set out in the OHRC with regard to protecting and respecting trans and gender-diverse students and was framed primarily in terms of ensuring accommodation that positioned the *individual* trans student as responsible for ensuring their own safety by requiring them to *request* accommodation (Omercajic & Martino, 2020). Unfortunately, what followed was a process of “policy borrowing” (Phillips & Ochs, 2004) from many other boards in Ontario, which have used this board’s policy as a template while others have relied on broader equity policies in which trans inclusivity is subsumed under a general equity focus.

The introduction of gender identity and gender expression into OHRC policy also resulted in the revision of specific policy stipulations for educational institutions, such as the Ontario Ministry of Education’s Policy/Program Memorandum 119 issued on April 22, 2013. This policy requires school boards to “develop and implement equity and inclusive education policies that address all forms of discrimination and harassment based on Code protected grounds, including gender identity and gender expression” (Ontario Ministry of Education, 2013). Although this provision resulted in the Ministry of Education updating its goals from the 2009 Equity and Inclusive Education Strategy with the Education Equity Action Plan highlighting gender identity and gender expression, specific and clear guidelines for supporting trans and gender-diverse students were not articulated (Ontario Ministry of Education, 2017; Martino et al., 2019). Kirkup et al. (2020) indicate that school boards play an important role in constructing a specific meaning of “gender identity” and “gender expression” that is not “expressly defined” in OHRC definitions (p. 248)—they dictate how such terms “are preventatively or reactively applied in school settings” (p. 261) that have not yet been seen in a legislative context. Notably, Omercajic and Martino (2020) demonstrated that these policies tend to rely on a prioritized discourse of accommodation over the need for proactive gender-expansive education that attends to questions of cis privilege and gender entitlement.

While there is evidence of a *hard policy*^5^ emphasis in Ontario, given the human rights legislation in the public sector, where the necessary grounds for anti-discrimination based on gender identity and gender expression are explicitly articulated by the Ontario Ministry of Education, our research reveals that this has not necessarily translated into adequate development of trans-inclusive policies by school boards or the enactment of these policies in schools. Moreover, it has certainly not translated into support and resourcing for PD and curriculum development committed to addressing the systemic impact of trans marginalization and need for gender-expansive education. For example, in 2015, the progressive Liberal Government of Ontario released a revised Health and Physical Education curriculum that mandated lessons regarding consent, cyberbullying, gender identity, sexual orientation, and contraception. While this updated curriculum was supported by provincial legislation (i.e., the Accepting Schools Act and Toby’s Act) “to increase [teachers’] comfort level and their skill . . . and to ensure effective delivery of the curriculum,” educators were expected to “seek out current resources, mentors, and professional development and training opportunities” on their own (Ontario Ministry of Education, 2015, p. 12). However, the election of the conservative party under Premier Doug Ford in 2018 resulted in its repeal. Ford mandated that schools revert to the previous 1998 Health and Physical Education curriculum while the government revised the 2015 document. The Ford government then launched a “snitch line” where parents could disclose if teachers were still teaching the repealed curriculum. This snitch line demonstrated the vulnerability of educators in the school system to government surveillance of their teaching in the face of parental and community backlash. One year later, Ford released an updated curriculum, delaying discussions surrounding gender identity from Grade 3 to Grade 8 despite the problems of the rationality about children’s developmental readiness driving these decisions, which had been documented in the existing literature (DePalma & Atkinson, 2006; Steele & Nicholson, 2020).

The introduction of gender and sexual diversity topics within the context of the Health and PE curriculum in Ontario incited a moral panic and media attention fueled by a parental rights discourse and a cultural politics of fear; these had the effect of containing a public discussion of these topics to this specific domain of curriculum, with its familiar mobilization of parental rights in response to sex education (see Morgan & Taylor, 2019). It produced a context in which teachers felt under siege, and directed attention away from and effaced how gender identity and gender expression are topics that need to be addressed across the curriculum. It meant that such topics became attached or *stuck* to an affective association with “sex” and a “cultural politics of fear” (Ahmed, 2004), leading to an incitement of parental rights being pitted against educating about gender and sexual diversity as fundamental human rights.

This backlash and pushback are significant because they draw attention to the politics of gender-expansive education and failure of hard-policy frameworks (K. Kennedy et al., 2011), which are grounded in human rights legislation, to ensure the provision of trans-inclusive rights—especially trans students’ rights to see themselves represented in the curriculum. Social conservatives were able to mobilize an insurrection against the progressive curriculum and to exert undue influence that was motivated by transphobic and cissexist beliefs about the capacities of children to understand and recognize themselves outside of cisgenderist and binary frames of reference (N. Kennedy, 2018; Steele & Nicholson, 2020). Hence, our research generates insights into educators’ understandings of policy within this polemic context to illuminate not only how the saturation of a particular policy discourse seeps into and suffuses understandings of trans-inclusive policies in the Ontario context, but also how such policy frameworks, with their emphasis on accommodation, expose a lack of accountability with respect to the necessary provision of curriculum reform that is committed to gender-expansive education for both educators and students in the school system.

## Theoretical Framework

We draw on trans theorists who bring a critical focus on cisgenderism, cisnormativity, and cissexism as central to our understanding of what it means to address trans marginalization in schools. All three terms provide a conceptual clarity in illuminating systems of oppression and unmarked privilege that are derived from assuming a gender identity aligned with that assigned at birth—a focus not encapsulated by a queer studies focus on heteronormativity (Radi, 2019). As Stryker (2006) notes, queer studies “fails to acknowledge that . . . transgender phenomena constitute an axis of difference that cannot be subsumed to an object choice model of antiheteronormativity” (p. 9; see also Martino & Cumming-Potvin, 2018; Martino & Omercajic, 2021). This antinormative focus, Chaudhry (2019) argues, has resulted in “transness” being invoked “as a category of potentiality, expansiveness, and diversity (in many ways as a kind of *ultimate* queer),” which not only “obscure[s] the often precarious realities for trans and gender nonconforming people (especially people of color) but also risk[s] further marginalization” (p. 47). Keegan (2020), thus, insists on a trans studies lens that specifically addresses trans people’s lived and embodied experiences of gender and elucidates the institutionalized systems of power that render their lives and identities intelligible in ways that are not afforded by a critical focus on “the dissolution of heteronormativity” and its “undergirding gender binary” (p. 391).

Central to our understanding of trans inclusion are questions related to gender privilege and erasure of trans identities, which reflect a broader institutional concern with how cisgenderism is institutionalized in school systems and governance structures that significantly impact both the development and enactment of policies committed to supporting trans and nonbinary students in the education system. Ansara (2010) defines cisgenderism as “the individual, social, or institutional attitudes, policies and practices that assume people with non-assigned gender identities are inferior, ‘unnatural’ or disordered and which construct people with non-assigned gender identities as the effect to be explained” (p. 190). However, N. Kennedy (2018) emphasizes the distinction between *cultural cisgenderism* and transphobia, which she defines as a

detrimental and predominantly tacitly held and communicated prejudicial ideology, rather than an individual attitude . . . [that] represents systemic erasure and problematizing of trans people and the distinction between trans and cisgender people . . . [and] essentializes sex/gender as biologically determined, fixed at birth, and externally imposed on the individual. (p. 308)

Although both “cisgenderism” and “cisnormativity” are often used interchangeably, Frohard-Dourlent (2016b) clarifies that the latter refers to “the belief that gender is a binary category that naturally flows from one’s sex assigned at birth” (p. 4). “Cissexism” is another related term, coined by Serano (2014) in her earlier trans activist work, and is used to distinguish between a universalized critique of the impact of binary gender norms, which also impacts cis people, and the specific form of gender oppression that affects transsexual people, who “challenge societal norms with regard to gender identity and sex embodiment” (para. 8). Hence, this term captures the ways in which the self-designated gender identities of trans girls/women and trans boys/men are often dismissed as illegitimate, and/or how trans girls/women and trans boys/men are treated “for supposedly being too conservative, assimilationist, or for reinforcing the gender [binary] system” (Serano, 2014, para. 8). Serano acknowledges that there “are advantages and limitations of different ways of employing cis terminology” but encourages strategic and productive use of such terminology “within a given situation” (para. 3). We find all these terms useful within the context of our critical examination of educators’ understandings of trans-inclusive policies because they help us to elucidate the institutional barriers that thwart the provision of support for trans students and gender-expansive education in schools.

However, Krell (2017) notes the importance of examining the elision of race and class in employing such terminology and how this contributes to reproducing “normative whiteness” and the “the implicitly racist and classist underpinnings of hard-line categories of sex and gender” (p. 235). This work thus draws attention to *racialized* cisgenderism, illuminating the ways in which antiblackness and settler colonialism have been sidelined in addressing trans marginalization in schools—an issue that we address in our analysis of educators’ understandings of trans-inclusive policies and their implications for gender-expansive education. For example, a cis-hetero patriarchal system of colonial imposition and domination is already embedded in imposed binary gender designated identities, which Driskill (2010) noted “open[s] up conversations about ongoing decolonial struggles and the relationships between sexuality, gender, colonization and decolonization” (p. 70). Morgensen (2012) too calls for interrogating this “settler epistemology as a social norm” that is at the heart of “the European establishment of Western heteropatriarchal and binary sex/gender systems in settler societies, as a condition of their religious, economic, and political life” (p.13). Hence, an understanding of the institutionalization and normalization of gender binary norms must be situated “within past and present formations of European settler colonialism” (Morgensen, 2012 p. 14), a point that we highlight is central to understanding trans-inclusive policy enactment and gender-expansive education in schools.

Our understanding of trans-inclusive policies is also informed by Spade’s (2015) articulation of *a critical trans politics*, which we apply to *administering gender* in the school system (Martino et al., 2020). Spade (2015) calls for a critical trans politics that “demands more than legal recognition and inclusion” (p. 1) and draws attention to how legal recognition in the form of anti-discrimination policies “on the basis of sex, race and disability” (p. 1) has not necessarily resulted in a more equitable “distribution of life chances” (p. 2) for trans people, particularly racialized trans people. Rather, he distinguishes between the law itself within the context of a settler colonial state, where “life chances are distributed through racialized-gendered systems of meaning and control” (p. 5), and its administrative enactment in institutions. We follow Spade (2015), who argues for the direction of critical attention to “administrative governance” of trans policies and, hence, to their deployment in schools with respect to addressing trans marginalization and the systemic impact of cisgenderism, cisnormativity, and cissexism. Hence, our focus is not so much on a critical analysis of power in the form of “individual/intentional discrimination” of trans students, but rather on learning more about what educators’ understandings of policy can reveal about “how the administration of gender norms impacts trans [students’] lives” (Spade, 2015, p. 73). As Spade notes, such a critical trans politics has the potential to illuminate “how administrative systems in general are sites of production and implementation of racism, xenophobia, sexism, transphobia and ableism under the guise of neutrality” (p. 73). Sumerau and Grollman (2018) provide additional insight into the “obscuring of oppression” (p. 322) specific to systemic patterns of racism and cissexism, and how individuals make sense of black and trans activism. They argue that investigating the “processes whereby people obscure oppression may become a powerful tool for understanding practices that maintain the persistence of social inequality and possibilities for social change” (p. 335).

In fact, Ahmed (2012) highlights specifically how “having a policy [may] become a substitute for action . . . (which can work to conceal the inequalities that make the law necessary in the first place)” (p. 11), thereby obscuring oppression and “the persistence of social inequality” for trans students in schools through invoking generalized and nonspecific appeals to embracing an ethic of inclusion and equality for all (Sumerau & Grollman, 2018). In this way, Ahmed draws attention to the ways in which an “investment in both law and policy” (p. 11) needs to be thought about in terms of its enactment, and how appeals to both can serve as merely performative gestures rather than translating into social action. She argues that a stated commitment to diversity and inclusion can in fact be “a way of not doing anything” (p. 121). Hence, Ahmed claims that we can learn a lot from practitioners about *institutional life* and how diversity is understood in ways that “allow[] racism and inequalities to be overlooked” (p. 14), particularly with respect to “what recedes from view” (p. 14) and is obscured in how educators make sense of trans inclusion in schools and the education system more broadly. Our study draws attention to how a commitment to trans inclusion and supporting trans students in schools is understood as a part of larger “equality regime” that serves to mask institutional processes that thwart a commitment to enacting gender and racial justice in schools.

## About the Study

This survey was part of an international study investigating educators’ understandings of policies and practices related to supporting trans and gender-diverse students in schools. It was developed through professional and community consultation and comprised 15 questions that inquired about participants’ familiarity and experience with trans-affirmative policy, knowledge of and comfort levels with vocabulary related to gender diversity and sexuality, and participant observations on barriers to supporting and affirming trans and gender-diverse students, as well as seven demographic questions. The survey was open to all educational workers in Ontario (e.g., teachers, administrators, educational assistants, or support staff working in Ontario schools) from October 2019 to March 2020 and was disseminated via social media. Educational affiliates (Ontario Secondary School Teachers’ Federation; Elementary Teachers’ Federation of Ontario; and Ontario Teachers’ Federation) supported this initiative by disseminating the survey through their online platforms as well. In all, more than 1,200 participants responded to the survey, and after the inclusion/exclusion criteria were applied, 1,194 surveys were analyzed. It is worth noting that large-scale survey research does not permit nuanced insight into specific board and school contexts, which have been the focus in our previous case study research (see Cumming-Potvin & Martino, 2018; Martino et al., 2020; Martino & Cumming-Potvin, 2019). This focus on context specificity was not the purpose of the study. However, it does reveal a glimpse into policy enactment and a proliferation of a policy discourse across a range of school board and school contexts, which speaks to the nature of the Ontario *policyscape* marked by a human rights legal requirement for all public institutions to address anti-discrimination on the grounds of gender identity and gender expression (Kirkup et al., 2020; Martino et al., 2019).

Participants were asked to share demographic information about gender identity, sexual orientation, racial/ethnic background, age, region, and professional position. They could choose to share as much or as little demographic information as they wished. A sweeping majority of respondents identified as Woman/Girl or Cisgender Woman/Girl (78%), and 7% identified as gender minorities such as Genderqueer, Gender-nonconforming, Non-binary, Trans Man/Boy, Trans Woman/Girl, Agender, Two-Spirit, Intersex, Genderfluid, or Not listed; these numbers are consistent with the Ontario College of Teachers 2020 report on the self-reported gender identity of members in good standing (Ontario College of Teachers, 2020). A total of 68% of respondents identified as Heterosexual/Straight. Among the remainder of the participants, 7% selected Bisexual, 5% Queer, 5% Pansexual, 4% Lesbian, 3% Gay, 2% Asexual, 2% Questioning, and 0.5% Demisexual. Participants mostly identified as White (84%), with the remainder of the sample indicating East Asian (3%); Black (3%); Indigenous (2%); South Asian (2%); Middle Eastern, Latin American, and Southeast Asian (less than 1%); and Other (5%) as their racial or ethnic background. A total of 89% of respondents were 55 years old or younger.

In this article, we focus on the questions devoted to educators’ awareness and knowledge of trans-inclusive policies. Educators were afforded a space to provide further comment on trans-inclusive policies in their school after responding to quantitative measures regarding their awareness of and administrative support for such policies. A total of 561 educators responded to this particular item in the survey. However, 29 comments were not coded and were deemed not classifiable (i.e., no comment/NA), and 69 were coded as “Other”; as a result, comments from 463 respondents were coded. Importantly, some participant responses were coded and applied to more than one thematic category, which accounts for the higher number of tallied responses in Table 1 (*n* = 510). Qualitative data from the surveys were analyzed following Braun and colleagues’ (2018) phases of thematic analysis. The research team began with a process of *familiarization*, spending hours individually and “closely read[ing] and thoroughly engag[ing] with the data” (p. 853). Beginning with a “bottom-up” inductive orientation, each team member reflected on the “meaning” within the data. This familiarization lent itself to a more “detailed and systematic engagement with the data” (p. 853) through a deductive approach informed by our reading of the theory and existing literature in the field; this allowed us to generate codes that were assigned to identify the issues being addressed by participants. To construct themes, an iterative process of collaboration ensured that similar codes were “collated, together with their associated data” (p. 855). This iterative process of collaboration led to depicting “a coherent insightful story about the data” (p. 855), given our research focus on learning more about educators’ understandings of trans-inclusive policies. We generated clusters of meaning both independently and collaboratively, “meeting regularly to discuss candidate themes” (p. 855) that were created through the phases of revising and defining, expunging any themes that risked “analytic ‘thinness’” (p. 855). We compiled all coded data for each of the candidate themes into a table that displayed each code next to candidate themes, allowing the team to review them “to ensure that the data relate[d] to a central organizing concept” (p. 855) and ultimately ensured that the themes “comprehensively and concisely capture what is meaningful about the data” (p. 857).

**Table 1.** Comments on Trans-Inclusive Policies in Schools.

Key Themes	Number of Comments
Policy as Accommodation	138
An Individualized Approach	64
Lack of Support/Intervention	114
Policy Versus Practice	109
Transphobic and Cissexist Resistance	22
Education and Curriculum	31
General Acceptance of Diversity	32

Overall, seven themes were organized to represent an overview of the findings (see Table 1). Given that thematic analysis “requires a degree of ‘theoretical knowingness’” (Braun et al., 2018, p. 857), our process of coding and identifying themes was guided specifically by the team’s trans-informed theoretical grounding and in Ahmed’s (2012) work on the politics of diversity and equality regimes in educational institutions. Ahmed, for example, noted that the experiences of practitioners in the education system can indeed offer us “the opportunity to “thicken” our description of institutions” (p. 8).

## Analysis of Data

Of the total number of respondents (*N* = 1,194), 73% indicated that they were either somewhat or very aware of their school board/school’s policy on supporting trans and gender-diverse students. However, 28% revealed that they had no awareness. A total of 94% found the policy to be very or somewhat relevant and indicated that they were likely to follow policy recommendations. Furthermore, 94% of respondents indicated that their school administration would be somewhat or very supportive of trans-inclusive and -supportive policies.

While these statistics provide a very favorable assessment of support for trans-inclusive policies in Ontario schools, the qualitative data depicted a very different reality. These findings highlight the discrepancy that exists between policy and practice (Bartholomaeus & Riggs, 2018; Taylor et al., 2016)—an issue addressed more broadly in the policy literature calling for research to investigate not only the policy itself but also its enactment in schools (Ball et al., 2012).

We organize our analysis by reordering the themes to better reflect and forge particular connections in our interpretation of the data rather than following the chronological order on the basis of frequency of response. We end our analysis with a critical focus on several comments from participants that were not coded according to any of the mentioned thematic categories but that raise important considerations about race and gender justice in education policy, given that they pertain to the need for an intersectional approach to addressing equity that avoids obscuring oppression (Sumerau & Grollman, 2018).

## Policy as Accommodation

A total of 30% of the educator comments reflected an understanding of trans-inclusive policy as accommodating trans students, where being inclusive meant providing gender-free washrooms, having GSAs, and using affirmed pronouns. A trans-inclusive policy was also associated with visible symbols, such as Pride or Rainbow flags being posted in schools. Many educators considered such interventions and practices as evidence of a commitment to trans-inclusive policies being enacted in schools and a measure of a high degree of support for trans students:

We finished adding our 4th gender neutral washroom in our building of only 2 floors to help make a safe washroom space be more accessible no matter where you are in the school.

We are encouraged to be aware of students’ choice of pronouns or names they prefer. We also have gender neutral washrooms for students that are single use washrooms.

Teachers can post the rainbow symbol on their doors to welcome students, staff, and parents. We have gender neutral washrooms.

This understanding of the trans-inclusive policy is not surprising, given the emphasis in many school board policies on accommodating trans students (Omercajic & Martino, 2020). Some educators noted the limitations of policy focused primarily on accommodation, highlighting the extent to which a commitment to accommodation merely translates into a surface-level support. They also indicated that simply providing an all-inclusive gender washroom space did not ensure that the needs of trans students were met:

We have an “all access bathroom” but you need a special key card to access it.

There is one bathroom in the high school designated for transgendered [sic] students. I am concerned that because this information is shared amongst the students that this may target this specific location as a focal point for bullying.

These statements exposed a degree of opaqueness (Ahmed, 2012) behind a commitment to trans inclusion that was articulated in terms of accommodating the needs of individual trans students, which we see as underscored by the limits of an emphasis in the policies themselves (Omercajic & Martino, 2020). Such a commitment failed to address the systemic forces of institutionalized cisgenderism at play, which led to intensified administrative surveillance and the potential risk of further policing and hypervisibilizing individual trans students (Bender-Baird, 2016; Cavanagh, 2010; Ingrey, 2018). Thus, rather than addressing trans marginalization and creating more livable conditions for trans and nonbinary students (Butler, 2004), such enactment of trans-inclusive policies exacerbated and increased the vulnerability of trans students, potentially subjecting them to further violence. Spade (2015) argued that policy and the law do not necessarily ensure that trans people are protected and that it is the very administrative systems responsible for enacting the policy that end up being “the greatest sources of danger and violence for tans people” (p. 16). Such a problem is further highlighted by an administrative approach to enacting policy that centers on the presence of the visible trans student in the school as evidence of a commitment to diversity and inclusion (Ahmed, 2012; Martino et al., 2020; Meyer et al., 2016). Thus, the pervasive incitement to a policy discourse and its enactment across many different school contexts through the eyes of many different educators is what our study illuminates.

## An Individualized Approach

A total of 14% of educator comments reflected an understanding of trans-inclusive policies as responding to individual trans students, or an individualized approach. Support and awareness of the policy were defined and understood in terms of the visible presence of a trans student who was out in the school. In many cases, the policy was considered irrelevant or not enacted in the absence of trans students declaring themselves. This approach resulted in an understanding of trans-inclusive policies being enacted on “a case-by-case basis”:

As of now it is **a non-issue** at our school, so it is **not applicable** as of yet. We have not had trans students, so awareness and policies have not been enacted [our emphasis].

I have not had the opportunity to be involved with a trans student at any of my schools to my knowledge. As such, I have not seen an administrative response to this situation, other than a general atmosphere of acceptance to the possibility.

We tend to approach on a case-by-case basis and build on what the student wants and needs.

As Ahmed’s (2012) work on *doing diversity* highlights, these statements enable us empirically to “thicken our description” (p. 8) of institutional life with respect to how trans inclusion policies are being taken up in schools. This individualized approach to trans inclusion absolves the system of accountability for addressing the deeper institutional forces of cisgenderism, cisnormativity, and cissexism at play that contribute to the erasure of trans students and that prevent them from coming out in schools in the first place (Youth Gender Action Project, 2009). As Frohard-Dourlent (2018) notes, such an approach relies on trans students (or their parents) “actively seek[ing] recognition of their identities in schools without institutional backing to do so” (p. 329). The result, as the following educators noted, means that trans inclusion is left to individual trans students and educators to initiate (Martino et al., 2020), with opportunities for education about gender expansiveness and what is needed to address the institutionalization of cisgenderist, cisnormative, and cissexist expectations and their embeddedness in settler colonial sex/gender binary systems simply effaced (Morgensen, 2012):

The school leaves it up mostly to individual educators but are supportive of any initiative that will foster a more inclusive learning environment.

We could do a better job as a school. I think that because we have no “visibly” trans students in our school our administration has not focused on this aspect of inclusion.

I feel that it is only addressed on a case-by-case basis. Rich conversations are not openly encouraged.

These educator accounts provide significant insights into how *institutional life* takes shape in schools with respect to how trans inclusion and diversity work are committed to supporting trans students (Ahmed, 2012). They highlight once again the effects of a particular policy discourse that is inextricably tied to a logics of accommodation.

## Lack of Support/Intervention

A total of 25% of the comments indicated a lack of support or intervention from administration, meaning that it was often up to the individual educator to enact the policy.

We are made aware of the policies, but it is left up to individual education workers to actually read and understand them. Attempts at implementing the policies are somewhat half-hearted. For example, use of gender-neutral pronouns has been attempted by only some staff, and not all of them continue such use.

Some educators indicated that they did not feel supported by the administration and expressed the need for more education. They claimed that the administration generally was simply ill-equipped to provide the support that educators and trans students needed.

Admin doesn’t really actively talk about these policies. The information we get comes from the school board. Any activities or events in support of trans-inclusiveness are initiated by interested staff. Admin neither actively supports nor has a negative view.

They [the principal], after several times that I have pointed this out, still have not fixed their system to print out class rosters with the students’ preferred name. This means that students can be outed when there is a supply teacher. This happens and until they’re brought before a human rights tribunal, it will continue to happen.

Principals rarely defend us, the teachers, on the front lines, boots on the ground, in the trenches [so to speak] to support us if there are any difficulties between a parent and a student because of our teaching and following the curriculum, policies and procedures.

Other educators commented that lack of intervention and support meant that not only was transphobic bullying not addressed in school, but individual staff had to take responsibility for educating themselves about trans inclusion. Such conditions resulted in the onus falling on the individual trans student to advocate for themselves:

Unfortunately, students are still bullied and attacked within schools and the policies do nothing to protect them. It’s hard for trans and LGBTQ students to feel safe when bullies face no consequences.

The reason I would not follow it is due to the lack of actual support the policy provides. There is zero consequence for transphobia and zero education for any staff members. I feel that our school is NOT supportive. Due to this I choose to look at outside resources to ensure support for my students.

We have a gender creative student. His teachers have had to seek help for educating themselves from outside the school. Washrooms are not inclusive. Sports teams continue to be gendered. People are not pro-actively educated, so this child must continuously self-advocate.

Both educators’ and administrators’ religious beliefs and/or biases were identified as barriers, leading to trans youth not being supported in schools and to a lack of significant intervention. Educators from Catholic schools were explicit about the lack of support in their schools, which they claimed resulted in trans students and gender diversity not being addressed, a contravention of the Ontario Human Rights Code. These educators highlighted the tension between the Catholic doctrine and provision of support for trans students in their schools:

The policy of my Catholic school board is to ignore and not mention trans students or employees. Although we are required to follow the Ontario human rights code, our school board hides behind the Catholic curriculum which only minimally addresses gender identity. Silence and actively dissuading teachers from speaking about gender is the real “policy” of our school board.

Our policy is currently under review. We have moved to having at least one gender neutral washroom in schools. We are still learning, as a district, to consistently balance the dignity of the student with the more challenging traditionalists of Catholic teaching who see this as an affront to their faith. Individual students are supported, but their existence as a group seems to challenge our school board.

We are a Catholic school and have had negative push-back from some parents regarding potential (open-minded and inclusive) discussions teachers might have when there is a child who identifies as non-binary in a class. We feel caught between following “Catholic Church doctrine” and teaching in a modern world. We always want to make all students feel welcome and accepted and loved.

Educators also attributed lack of support and intervention to administrators’ lack of understanding of trans inclusion and gender diversity:

Our administration does not understand the difference between transgender, non-binary and non-gender-conforming; there is no attempt to use gender-neutral language; no discussion about gender-neutral washrooms for children.

Teachers understand board policies. There is a disconnect with administrators, who do not seem to understand these policies.

The principal is very binary in all situations, even situations that could easily be modified, like giving out 2 awards, vs. giving 2 awards (one male and one female).

As one participant pointed out, simply lumping trans students under the “LGBT or queer community umbrella” masks and obscures the “widespread discrimination and exclusion against people of trans experience” and “affirmed genders” (Ansara, 2010, p. 187):

Trans issues are being categorized under the umbrella of LGBTQISS+++ and this does not serve trans students’ needs enough. There are trans specific issues such as gendering students and bathrooms that are not being “mandated” or “regulated” or even talked about.

While existing research has pointed to lack of support for trans inclusion and gender-expansive education, our data afforded further insight into this problem in both publicly funded secular and Catholic school systems in Ontario. Educators here drew attention to how individual beliefs in both school systems serve as impediments to enacting trans-inclusive policy and gender-expansive education and how these are further exacerbated by more macro-level influences in the Catholic school system (Campbell et al., 2021; Herriot & Callaghan, 2019; Taylor et al., 2016).

## Policy Versus Practice

Our findings highlighted a gap between policy and practice, revealing that “what happens inside a school in terms of how policies are interpreted and enacted [are] mediated by institutional factors” (Ball et al., 2012, p. 10) and administrators’ lack of support in the provision of resources. One in five respondents (24%) commented that simply having a policy does not mean that it will be followed, and many provided insight into how both the context and interpretation affect how policy is translated into practice:

As a supply teacher, I work in many schools, so the policies of the school board are manifested in different ways across the schools. Some administrators support and implement trans inclusive policies more than others.

My admin is somewhat supportive but afraid of parent backlash. I think a lot of their steps forward though are due to my presence as an out queer teacher who is very vocal about upholding policy

As discussed earlier, this concern about backlash, which was expressed by numerous educators, has a particular salience in the Ontario context; it speaks to the residual effects of a moral panic that irrupted in response to the introduction of gender and sexual diversity topics in the health and PE curriculum in Ontario in 2015 that was fueled by a parental rights discourse and a cultural politics of fear.

Educators specifically pointed out that one’s own beliefs, prejudices, and biases come into play and impact policy enactment; even when there is widespread support, there is a lack of accountability:

While many of my colleagues are supportive, the several who are not are very vocal and have the ear of our admin. As such, there is unlikely to be any school-wide acknowledgement of trans-inclusive policies and/or the LGBTQ+ community at large.

Teacher and admin’s personal religious beliefs often bias their actions when working with trans students. While they may follow policy they do so very reluctantly and loudly complain about it.

There are general inclusion policies and best practices shared, but no real accountability and no follow up for teachers who don’t really follow them, or even worse, sabotage these.

They also drew attention to the problem with what Spade (2015) refers to as “administrative systems” in creating further vulnerability and surveillance of trans students in the provision of access to designated all-inclusive washroom spaces in the school. They pointed out that merely having a policy that supports gender-inclusive washrooms does not mean that trans students are going to be served well by creating these inclusive spaces:

They recently added an all-gender bathroom, but you have to go to the office to get a pass key to use it, so it singles people out that way.

There is some pushback as to the organization and management of the gender-neutral bathrooms. No one is denied access but some kids have taken advantage of it, caused some damage, general mischief etc.

These responses draw attention to the broader systemic impact of cisgenderism and its institutionalization in schools (Frohard-Dourlent, 2018), which is manifested here, both in terms of the administrative system of surveillance, which results in hypervisibilizing the trans student under the regulatory gaze of office staff, and with respect to how easily such spaces get colonized and violated by cis students (see Omercajic, 2022). These responses also highlighted the need for administrative policy support. However, for some educators, the policy was only relevant in spaces where there were openly trans students, which once again points to the problem of prioritizing a policy discourse that is driven by a logics of accommodation—particularly given the absence of a more educative focus on understanding the systemic forces of cisgenderism, cisnormativity, and cissexism in the school system and the extent to which they are embedded in a settler colonial logics (Morgensen, 2012).

While administrators may consider the creation and provision of access to washroom spaces as evidence of their support for trans inclusion, educators specifically identified their lack of support for and understanding of trans inclusion:

Board initiatives are broadly misinformed and led by individuals who do not understand the issues. Principals often resist involvement or support and provide only superficial window dressing support when forced to do so.

The trans-inclusive policy was not enforced last year. Our vice principal allowed students to yell hate speech down the halls without any repercussions.

Even though the school board has trans-inclusive policies, the school administration refuses to even mention anything about our transgender student, and cancels the international day against homophobia and transphobia every year due to “everyone is so busy.”

Educators also identified the elementary school context, where erasure and vulnerability of trans students are particularly palpable, as a particular problem:

In a K–6 school, we have rarely encountered gender creative students. Having said that, I have found preparation for nonbinary gendered students to vary in regards to bathroom use from principal to principal.

Currently have a 5-year-old expressing she wants to be a boy. In the elementary level teachers and administrators are ill equipped. Nothing is in place.

I feel that not enough of these conversations and awareness is occurring in the elementary schools as it appears that adults/parents are afraid that the students are too young to be exposed to these conversations.

The concerns about children “being too young to be exposed to these conversations” help to explain the specific barriers at play in the enactment of trans-inclusive policies in elementary schools (see Campbell et al., 2021, p. 985). These statements echo past claims about children not being developmentally ready to learn about and understand gender and sexual diversity, which rely on problematic constructions of childhood innocence (DePalma & Atkinson, 2006; Robinson, 2008):

We are a K–6 school so some teachers often think of trans-inclusive policies as premature.

I work with really young children. This is not an important issue for this age group. Reading, writing and math or more important.

A number of educators noted problems with the policy itself, highlighting that some policy texts “offer limited possibilities for interpretation” and action, which foregrounds once again the limits of such a reception regime that is delimited by a human rights focus, with its ensuing emphasis on individualism (Ball et al., 2012). As the following comments reveal, such policies do not address the systemic barriers related to the institutionalization of cisgenderism, cisnormativity, and cissexism that are at the heart of trans marginalization and create conditions of vulnerability for trans students in the education system (Bartholomaeus & Riggs, 2018; Omercajic & Martino, 2020; Phipps & Blackall, 2021):

I don’t think that the policies really look at the barriers which trans folks face. The policies are not about uncovering and challenging biases.

Attitudes are supportive but real practical ways of aiding students’ mental emotional well-being is lacking.

## Transphobic and Cissexist Refusal of Trans Inclusion

A notable minority of respondents (5%) outrightly rejected trans inclusion and expressed a degree of transphobia and cissexism that was troubling. Some educators citied familiar discourses identified earlier about childhood innocence and lack of readiness of children to grasp an understanding of gender diversity, which are in contradistinction to the existing literature about gender-creative and independent children (Ehrensaft, 2016; Pyne, 2014):

There are only two genders. Let’s stop damaging children by lying to them about SCIENCE. We’re supposed to be educators for heaven’s sake!!!!

2 scoops, 2 genders, 2 terms. MAGA.^6^

This should be **age appropriate**. A 5-year-old is just as likely to identify as a cat as she/he is a different gender [our emphasis].

This shouldn’t be something in elementary schools. Back in the day girls were tomboys and didn’t need to identify as being questionable. Now students are confused and think that it’s cool.

Half our PD is about this stuff. We should be learning about pedagogy with the taxpayers’ money.

Catholic faith supports specific male and female gender roles as defined by our DNA.

[I so do] not agree that teachers, students, and parents are forced to accept and teach an ideology that contradicts their faith. This is morally and ethically wrong.

While this group was extreme in their outright refusal of trans lives, their readiness to make public their transphobic views suggests that, between them and the majority participants who expressed their support through this survey, there exists a vast array of educators who harbor similar sentiments but do not outwardly voice them. In this context, the nature of these responses raised concerns about the harm of such beliefs and the extent to which respondents lacked knowledge and understanding about gender diversity. As Britzman (1998) indicated, such ignorance is not so much about a lack of knowledge as it is an active refusal of it, which has clear implications for understanding the impact of cisnormalization in maintaining the status quo and the current sex/gender polarized system that has its legacy in a settler colonial ontology, as Morgensen (2012) notes.

### Education and Curriculum

Educators indicated gaps in the policy related to the focus on accommodation and the need for more education on how to address gender diversity as part of the curriculum. Their comments demonstrate how a lack of education impacts the ability to enact the policies in ways that serve trans students and lead to creating a more gender-inclusive culture in schools:

We have two gender diverse children in our school that I know of, and we have NO education or discussion about how to engage or educate regarding the policies.

I believe that education for all educators around gender and transgender students needs to be completed and revisited frequently as our understanding and body of research grows.

I’m a supply teacher so see a lot of rainbow flags and sometimes gender neutral bathrooms, but many schools stop there and don’t include trans content in their books and curriculum.

These comments reflect that while educators may be supportive, they lack the knowledge and skills to adequately support trans students, highlighting both the “gap between goodwill and action aimed at proactively ensuring the inclusion of transgender people in schools” and the need to understand that “supporting individual students is different from challenging the inherent cisgenderism in schools which is needed for lasting change” (Bartholomaeus & Riggs, 2018, p. 141). Moreover, these comments echo the findings of Campbell et al. (2021), who note that “if educators are confident in their level of school support,” then they are more inclined to “find ways to incorporate 2SLGBTQ+-inclusive practices, even in religious contexts or Catholic schools” (p. 985).

However, educators overall in the study called for *mandated* PD, pointing to the policy failure and lack of system-level accountability for the provision of gender-expansive and trans-affirmative education:

Colleagues “who are not aware” or open, need to be educated or mandated to learn that’s the role of admin.

Have mandatory training for all staff on how to be more trans-inclusive.

Make the learning mandatory for ALL publicly funded professionals and ensure that all schools have safe spaces identified so that the conversations can start happening.

Mandatory PD for teachers and school administrators as well as having the topics included in the Ontario physical health curriculum [are needed].

Policies are supported but resources and PD to help educators navigate how to put forth practical ways of supporting students is much needed.

In calling for mandatory PD and the provision of resources to support their learning, educators are demanding *a more binding policy approach* to ensuring the development of “curriculum resources and support materials,” “the provision of school-based support to curriculum development,” and “creating time and space for teachers and learners” (K. Kennedy et al., 2011, p. 48). These comments highlight the system-level failure of trans-inclusive policies in the Ontario context that renders them largely ineffective and “soft” in the absence of budgetary allocation. In fact, educators drew attention to how such policies actually created conditions that further exacerbated trans marginalization in schools and thwarted teachers’ efforts and commitment to supporting trans students, often resulting in individual trans students and their parents having to advocate for themselves (Carlile & Paechter, 2018; Ferfolja & Ullman, 2021; Martino et al., 2020).

## General Acceptance of Diversity

Some educators comments (7%) indicated that they considered their schools to be inclusive regardless of whether there was an explicit trans-inclusive policy, or they simply equated trans inclusion with a liberal embrace and celebration of diversity:

We don’t go out of our way to recognize specific individuals as unique, but our policy is to be inclusive of all students.

There’s an awareness concerning it, but as it’s a K–5 school, it follows more along the process of equity, inclusion and awareness of bullying for whatever reason.

I don’t think they have a straight up policy; I think it is really just we live in a day and age where it doesn’t matter who you are and everyone gets treated the same.

However, as Marx et al. (2017) note, “treat[ing] all students the same . . . willfully ignores the differences that may in fact require [one] to see and acknowledge a trans student as differently impacted by gender norms” (p. 1; see also Frohard-Dourlent, 2016a).

Overall, educator comments highlight the problem of resorting to a general approach to addressing equity, obscuring and deflecting attention from the specific systemic barriers that contribute to trans marginalization. Bartholomaeus and Riggs (2018), for example, point out that cisgenderism is responsible for binarizing, misgendering, erasing, pathologizing, and marginalizing trans and nonbinary students in schools, while Marx et al. (2017) argue that “appealing to the ‘sameness’ of ‘all students’ reinforces cisnormativity” (p. 11). Such approaches fail to effectively serve the needs of students who have multiple and intersecting identities. For example, a general approach does not foster knowledge about interlocking systems of oppression in the lives of Two-Spirit people, Black people, people of color, and those from various faith backgrounds who are trans, nonbinary, and gender diverse (Laing, 2021; Muslim Youth Leadership Council, 2019; YouthLine, 2020a, 2020b).

Equity frameworks that subsume all minority and racialized groups under a generalized embrace of diversity and inclusion obscure how settler colonialism, anti-Black racism, ableism, cisgenderism, heterosexism, and classism intersect in the lives of students from different communities. As Sumerau and Grollman (2018) have highlighted, such an approach “combines individualist discourse” with a liberal embrace of diversity, which effaces “the ways that covert forms of racism and cissexism may find voice in relation to racial and gendered others” (p. 323). Moreover, Ahmed (2012) elucidated how appeals to such generalized approaches illuminate a particular *grammar of diversity* that is employed by educators in our research to understand trans inclusion in ways that function to obscure oppression in the lives of trans students in schools. Our analysis demonstrates how generalized appeals to diversity and inclusion provide insight into how a commitment to trans inclusion surfaces in the Ontario school policy context and how cis white privilege “recedes when diversity comes into view” (Ahmed, 2012, p. 185). Hence, educator “statements of commitment can thus be read as opaque . . . insofar as they do not describe what is being done” (pp. 116, 120) to support trans students beyond merely claiming a generalized support for all students.

## Race and Gender Justice in Trans-Affirmative Education Policy

In this section, we discuss several uncoded comments in which respondents expressed the necessity of focusing on First Nations, Métis, Inuit, or immigrant students in their schools, which they indicated meant that attention to trans inclusion could not be addressed or that it was simply not a topic that had “come up”:

In a First-Nation private school, it is hard to balance self-determination and sovereignty of First Nations against imposing values such as trans-rights in some communities which do not have a history of individualized values.

My administrators will support any policy put forth by the board. My board is currently pushing FNMI [First Nations, Métis, and Inuit] content, so the policy on trans-inclusivity is present and accommodating but not pushed.

This topic has not really come up in my school. We have a huge Syrian refugee population and extreme violence in our school with students of needs. Our priorities are surviving from one day to next without injury to all.

The first educator erroneously equates trans inclusion with an *individualized* focus on human rights that is imposed and that is contrary to the ethic of self-determination and sovereignty of First Nations, Métis, and Inuit peoples. Spade (2015) is clear that such an “an individual rights” framework, as it is structured in legal systems, has served to “create a racialized-gendered distribution that perpetuates violence, genocide, land theft, and exploitation” (p. 7). This comment highlights the need to take into account the intersectional experiences of youth in any community and points to an overall failure of the existing trans-inclusive policy framework. It is also important to understand that issues of violence experienced by refugees are also further compounded for those who experience homophobia and transphobia as queer and trans people and who are erased when the mentioned educator refers to Syrian refugees.

As such, the educator comments illuminate the necessity of addressing interlocking and intersecting systems of oppression in students’ lives, with attention being paid to supporting 2SLGBTQ+ Indigenous and immigrant youth in schools. They reveal the siloed thinking that governs and prioritizes equity needs within a school board and the need for a critical trans politics that “interface[s] with anti-racism, feminism, anti-capitalism, anti-imperialism, immigration politics and disability politics” (Spade, 2015, p. xiv). The effect of such silos is to efface and erase other differences within these communities. For example, 50% of Indigenous 2SLGBTQ+ and 58% of racialized 2SLGBTQ+ youth across Ontario indicated that they did not “feel a sense of community” (YouthLine, 2020a, pp. 2–3) and called for more visibility and representation in their schools and communities, with the former calling for “2S spaces” where they can “connect with all of [their] identities” (YouthLine, 2020b, p. 2). As Nicolazzo (2021) highlights, “[j]ust as trans* people need physical space to be themselves, we also need epistemological spaces of our own to learn how we come to know ourselves and our worlds through gendered perspectives” (p. 517). These educators additionally reveal an affective sense of being overwhelmed, which is the product of working in a system with ever-expanding and complicated demands that lead to equity issues being treated and understood in siloed ways. Their prioritizing of “survival” points to conditions in schools where educators, lacking the necessary human and material resources, as well as school governance that works to make intersectional oppression visible, have no means to undertake this work individually.

## Implications and Conclusion: Addressing Policy Failure and the Need for Accountability

In this article, we have provided empirical insights into the gap between trans-inclusive policy and practice in schools, and, more significantly, into how a lack of administrative support for the enactment of these policies, combined with educators’ understandings of them, contributes to an exacerbation of trans marginalization in schools by obscuring the very systems of oppression that contribute to it (Frohard-Dourlent, 2016b; Martino et al., 2020; Marx et al., 2017). More specifically, we have shed light on the extent to which many educators trade on a logics of trans inclusion and liberal individualism in making sense of these policies, with the legitimating effect of deflecting attention away from a systemic commitment to the provision and resourcing of trans-affirmative and gender-expansive education. In short, we expose how invoking a discourse of accommodation allows for the legitimation of a policy failure and a lack of accountability that relies on self-responsibilization on the part of trans students to ensure their own recognizability and well-being in schools. In fact, we generate knowledge about the *doing* of trans-inclusive policies from the standpoint of many educators in Ontario. Our study exposes that while legislative frameworks are indeed necessary, they remain largely ineffective or “soft” because of the failure of policy makers to provide more binding policy instruments and budgetary allocation in order to ensure the provision of curriculum resources, support materials, and PD for educators in schools (K. Kennedy et al., 2011). Hence, we advocate for a shift in policy making away from an emphasis on a trans-affirmative discourse of inclusion and accommodation to a trans-*affirming* embrace of gender expansiveness conceived of as an ongoing, dynamic project of gender justice in schools—one that requires sustained attention to resourcing PD and curriculum development. The theoretical resources that we drew on assisted us in making sense of such phenomena, particularly in understanding both the policy and cultural context in Ontario and Canada. As Ahmed (2012) has noted, a commitment to institutionalizing diversity has become an officially sanctioned goal for organizations, but remarked that this does not mean that “an institutional aim to make diversity a goal” (p. 23) translates into an enactment of such a goal.

While scholars in the field have called for “a deeper paradigm shift towards *applying queer and anti-oppressive pedagogies* [emphasis added] in Pre-k–12 schools” (Meyer at al., 2016, p. 39), we argue that such a shift requires a necessary engagement with trans studies as a basis for addressing trans marginalization in schools (Martino, 2022). A critical trans pedagogical focus on cisgenderism, cisnormativity, and cissexism is required, which necessitates the systemic provision of and support for PD as inextricably tied to its realization—a commitment that has not always been adhered to in lieu of relying on a queer antinormative politics in conceiving of gender justice reform in schools (Martino & Cumming-Potvin, 2018; Martino & Omercajic, 2021). As Radi (2019) states, trans studies provides “tools for interpretation that, for the first time allow us to grant meaning to a number of previously unclassifiable collective experiences” and identifies cis privilege and cissexism as “crucial concepts in making sense of experiences of [trans] marginalization” (p. 54). These tools are pivotal to envisioning the paradigm shift that is necessary to improve the conditions in schools for trans students.

Furthermore, in conceiving of a much-needed paradigm shift in envisioning a more trans-affirming policy and its enactment in schools, we want to continue to draw attention to the limits of a queerly informed commitment to dismantling a heteronormative gender binary system. Such a framework for gender justice reform fails to understand the specific lived and embodied experiences of children and youth in schools with nonassigned gender identities (Ansara, 2010) and tends to rely on reinstating binary oppositional frames of reference. For example, a revisioning of both trans-inclusive policies and education in schools and educational research is needed that avoids imposing an oppositional “binary” and “nonbinary” trans nomenclature. This interpretive frame fails to account for how trans youth themselves choose to self-identify in ways that confound such a binary categorization (see Bower-Brown et al., 2021; Martino et al., 2020). Such a critical trans focus raises vital questions about the need for a commitment to trans desubjugation (Herriot & Fry, 2021; Stryker, 2006) in both the development and ongoing enactment of trans-affirming policies and gender expansive education in schools, as well as in educational research that is committed to creating conditions that support the livability of trans youth and gender democratization in the education system.

## Notes

1. We use “transgender” and “trans” interchangeably throughout the article and understand them as umbrella terms that encompass many gender identities of those who do not identify with their birth-assigned sex. We do not associate the concept of transgender with gender expression, sexual orientation, physical anatomy, or how one’s identity is perceived by others.

2. All three terms provide nuanced insight into the unmarked privilege, discrimination, and oppression that result from the legitimation and normalization of birth-assigned gender. We elaborate on an understanding of each of these terms later in the Theoretical Framework section.

3. As Kirkup (2021) noted, the Charter of Rights and Freedoms covers a “broad range of rights,” while the Canadian Human Rights Act addresses only “equality rights” in federal jurisdictions.

4. Specifically, in Ontario, both secular and Catholic schools are publicly funded and retain significant autonomy over curriculum and policy.

5. K. Kennedy et al. (2011) defined “hard policy” implementation as one that is characterized by “legally binding regulations” (p. 43).

6. Acronym for “Make America Great Again,” a political slogan used by Donald Trump during his American presidential campaigns.
